# ﻿Lectotypification of *Cycasdebaoensis* (Cycadaceae)

**DOI:** 10.3897/phytokeys.211.93650

**Published:** 2022-10-14

**Authors:** Yong Yang, David K. Ferguson

**Affiliations:** 1 Co-Innovation Center for Sustainable Forestry in Southern China, Key Laboratory of State Forestry and Grassland Administration on Subtropical Forest Biodiversity Conservation, College of Biology and the Environment, Nanjing Forestry University, 159 Longpan Road, Nanjing 210037, China Nanjing Forestry University Nanjing China; 2 University of Vienna, Department of Paleontology, 1090 Vienna, Austria University of Vienna Vienna Austria

**Keywords:** Cycadaceae, *Cycasdebaoensis*, gathering, gymnosperm, lectotypification, nomenclature, Shenzhen Code

## Abstract

The type of *Cycasdebaoensis* Y.C.Zhong & C.J.Chen (*Y.C.Zhong 8762*) consists of nine duplicates in PE. Our new investigation of the type collection suggests that the type includes at least two different gatherings which should be considered as syntypes. For nomenclatural purposes, we lectotypify the name *Cycasdebaoensis* with the herbarium sheet PE00047578 and consider other duplicates in PE and GXMI as isolectotypes. The seeds in the capsule (*Y.C.Zhong s.n.* collected in Oct 1998) are considered as a syntype.

## ﻿Introduction

*Cycasdebaoensis* Y.C.Zhong & C.J.Chen belongs to the gymnosperm family Cycadaceae. This species is similar to *C.multipinnata* C.J.Chen & S.Y.Yang in the bi- or tri-pinnate compound leaves, but differs from the latter by its shorter and more numerous leaves with narrower, thicker leaflets and the longer megasporophylls with a larger lamina (Hill 2008). It has extremely small populations occurring in Guangxi and Yunnan of China (Chen and Zhong 1997; Chen and Stevenson 1999; Xi et al. 2022) and is considered as Critically Endangered (CR), (Hill 2008; Yang 2021). The species is listed as a first class species in the recently released National Key Protected Wild Plant Species of China (http://www.forestry.gov.cn/main/3954/20210908/163949170374051.html).

*Cycasdebaoensis* was formally described by Chen and Zhong (1997) and *Y.C.Zhong 8762* deposited in PE and was designated as the type specimen. We made an investigation of the type specimen and discovered that the situation is far from straightforward. There are nine herbarium sheets of *Y.C.Zhong 8762* in PE. Of these nine sheets, three have labels in Y.C.Zhong & C.J.Chen’s handwriting (PE00047578, PE00047579, PE00031036), while the other six possess only printed labels (PE01458925, PE01458924, PE00934025, PE00934026, PE00934027 and PE00934028). The labels of these duplicates indicate the type status (holotype or isotype): seven of them are marked as holotype/holotypus, while two of them are labelled as isotypus. Although Lin (2014) considered that the duplicate (PE00934026) is the holotype, this is not the case.

The nine sheets cannot be considered as a gathering. There are no serial numbers showing that all of them belong to a single gathering: six of them have information on the collection labels indicating that they are parts of a gathering, i.e. 1/9 (PE01458925), 2/9 (PE01458924), 3/9 (PE00934025), 4/9 (PE00934026), 5/9 (PE00934027), 9/9 (PE00934028), but three others only possess handwritten collection notes and are lacking such information. Moreover, one of the nine duplicates (PE00031036), in fact, consists of two different parts: the leaves and the megasporophylls were collected in Aug 1997 (*Y.C.Zhong 8762*), whereas the seeds in the capsule were collected in Oct 1998 (*Y.C.Zhong s.n.*). Under Art. 8.2 of the Shenzhen Code (Turland et al. 2018), the term “gathering” is used for a collection presumed to be of a single taxon made by the same collector(s) at the same time from a single locality. The type of *C.debaoensis* clearly includes two different gatherings that constitute syntypes. Under Art. 8.1, a type should be a specimen, namely a gathering or part of a gathering; we thus think that the name *C.debaoensis* requires to be lectotypified.

The duplicate (PE00047578) bears the authors’ handwriting indicating “*Cycasdebaoensis* Y.C.Zhong & C.J.Chen sp. nov.” and “holotypus”, suggesting that the specimen was studied by the original authors. Two other duplicates (PE00047579, PE00031036) also bear handwritten identification labels which, however, are marked “isotypus”. Moreover, the specimen PE00047578 is well preserved and includes both leaf characters and female reproductive characters (three megasporophylls with five young seeds attached to one of the megasporophylls). We thus designate *Y.C.Zhong 8762* (PE00047578) as the lectotype here and consider the other duplicates as isolectotypes.

## ﻿Typification

### ﻿ Cycas debaoensis

Taxon classificationPlantaeCycadalesCycadaceae

Y.C.Zhong & C.J.Chen, Acta Phytotax. Sin. 35(6): 571 (1997).

6CAD6E60-F945-5DB2-AF58-A3B4EAD754BA

Figs 1 , 2

#### Type.

China (中国). Guangxi (广西), Debao Co. (德保县), rocky mountains, alt. 850 m, brown calcareous soil, near huge rocks, height 2.9 m, leaves compound 3-pinnate, female with young seeds, 27 Aug 1997, *Y.C.Zhong* (钟业聪) *8762* (lectotype: PE00047578, here designated; isolectotypes: PE00047579, PE00031036 excl. seeds in the capsule collected in Oct 1998 by Y.C.Zhong, PE00934025, PE00934026, PE00934027, PE00934028, PE01458924, PE01458925, GXMI050022).

**Figure 1.**
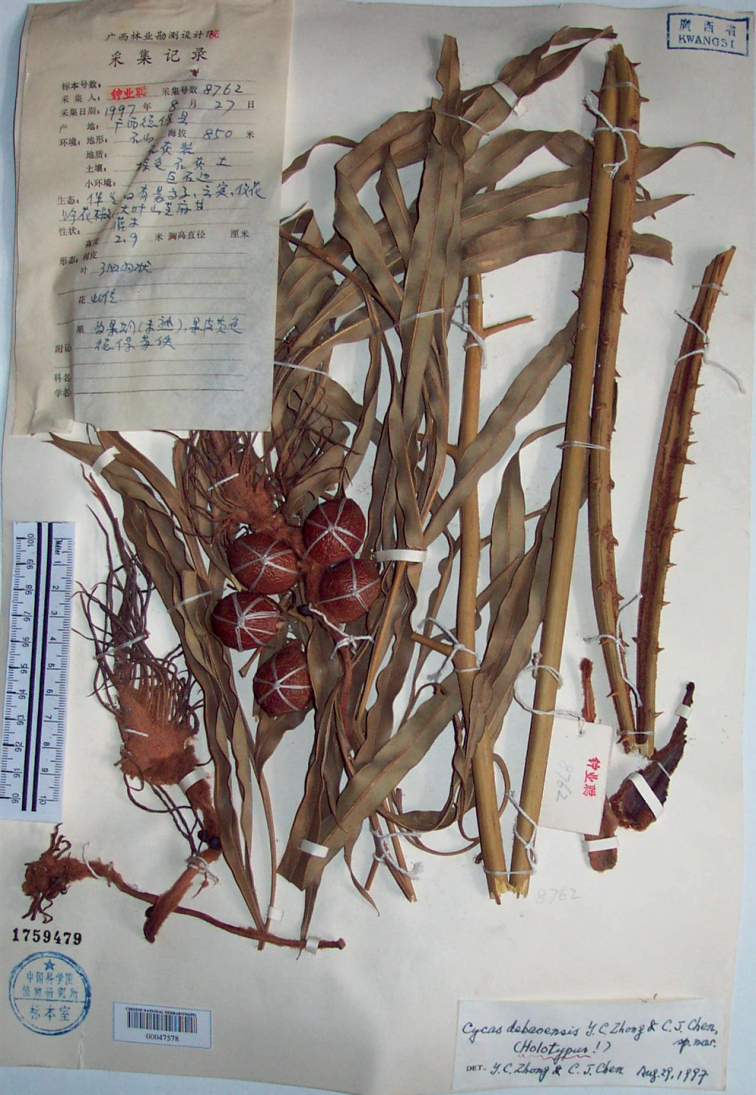
Lectotype of *Cycasdebaoensis* Y.C.Zhong & C.J.Chen: *Y.C.Zhong 8762* (PE00047578).

#### Note.

The seeds in the capsule of the duplicate (*Y.C.Zhong s.n.*: PE00031036) should be considered as a syntype. The protologue and type specimen images are available in Suppl. material 1.

**Figure 2.**
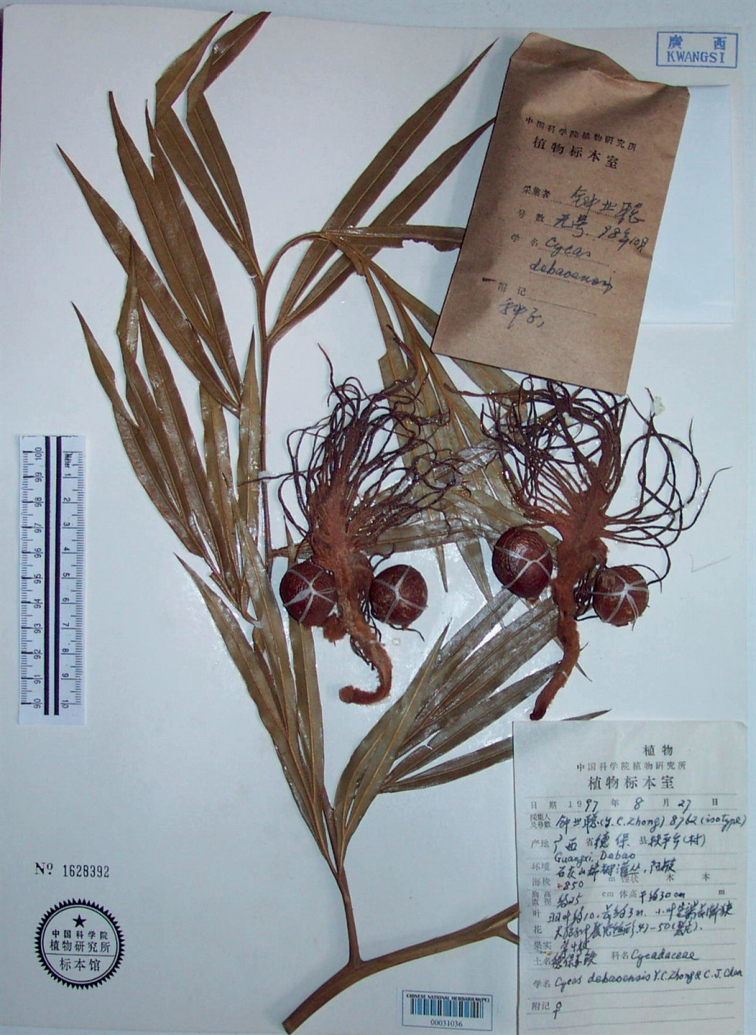
Two different gatherings mounted on a single herbarium sheet (PE00031036): leaves and megasporophylls (*Y.C.Zhong 8762* collected in Aug 1997) and seeds in the capsule (*Y.C.Zhong s.n.* collected in Oct 1998).

## Supplementary Material

XML Treatment for Cycas debaoensis

## Supplementary materials

Supplementary material 1 Figures S1–S11 Data type Figures (in rar. archive) Explanation note Isolectotypes and protologue of *Cycasdebaoensis*. https://binary.pensoft.net/file/756901This dataset is made available under the Open Database License (http://opendatacommons.org/licenses/odbl/1.0/). The Open Database License (ODbL) is a license agreement intended to allow users to freely share, modify, and use this Dataset while maintaining this same freedom for others, provided that the original source and author(s) are credited.Yong Yang, David K. Ferguson
